# LncRNA H19 induces immune dysregulation of BMMSCs, at least partly, by inhibiting IL-2 production

**DOI:** 10.1186/s10020-021-00326-y

**Published:** 2021-06-15

**Authors:** Xinpeng Chen, Xiuxia Luo, Yazhi Wei, Hualin Sun, Liping Dai, Yidou Tangzhou, Huijie Jin, Zhihua Yin

**Affiliations:** Rheumatology Department, Shenzhen Futian Hospital for Rheumatic Diseases, Nonglin Road 22#, Futian District, Shenzhen, 518040 Guangdong China

**Keywords:** Systemic lupus erythematosus, lncRNA H19, IL-2, Immune regulation

## Abstract

**Background:**

Systemic lupus erythematosus (SLE) is a representative systemic autoimmune disease. LncRNA H19 has been identified to participate in various biological processes in human diseases. However, the role of H19 in SLE remains unclear.

**Methods:**

In this study, we first examined H19 expression in SLE patients by RT-qPCR and found that H19 expression was significantly upregulated in the serum and bone marrow-derived mesenchymal stem cells (BMMSCs) of SLE patients and positively associated with SLE disease activity index. We then performed gain-of-function and loss-of-function using mimic-H19 (H19-OE) and inhibitor-H19 (H19-KD) to examine the effects of H19 on BMMSC differentiation, proliferation, migration, and apoptosis using flow cytometry, DAPI staining, and migration and apoptosis assays.

**Results:**

The results showed that H19 inhibited proliferation and migration but promoted apoptosis of BMMSCs, interfered with BMMSCs-mediated Treg cell proliferation and differentiation, and regulated BMMSCs-mediated Tfh/Treg cell balance. Dual-luciferase reporter assay confirmed the in silico prediction of interaction between H19 and IL-2. Furthermore, RT-qPCR showed that H19 directly inhibited IL-2 transcription in BMMSCs. ELISA showed that both active and total IL-2 protein levels were significantly lower in SLE BMMSCs. More importantly, we found that IL-2 significantly enhanced H19-OE-induced Treg cell differentiation and migration of BMMSCs, and these effects were reversed by anti-IL-2 antibody.

**Conclusion:**

Overall, our study indicates that LncRNA H19 induces immune dysregulation of BMMSCs, at least partly, by inhibiting IL-2 production and might be a novel therapeutic target for SLE.

## Introduction

Systemic lupus erythematosus (SLE) is a chronic autoimmune disease with diverse manifestations and clinical course characterized by disturbed T cell homeostasis (Wieliczko and Matuszkiewicz-Rowinska 2017). The incidence of SLE has been variably estimated to be approximately 1–10 per 100,000 people every year worldwide (Danchenko et al. 2006). With the progress of science and technology, early diagnosis, and better management from the 1950s to 2000s, the overall 5-year survival and 10-year of SLE patients have significantly increased from 74.8 to 94.8% and 63.2 to 91.4%, respectively (Mak et al. 2012). Despite these advantages, the management of SLE patients still faces significant challenges. Hence, understanding its specific molecular mechanisms will contribute to identify and develop specific therapeutic targets for SLE.

Autoimmune diseases represent a family of at least 80 illnesses that share a common pathogenesis: an immune-mediated attack on the body's own organs (Rose 2016). Bone marrow-derived mesenchymal stem cells (BMMSCs) originally isolated from the bone marrow stroma are multipotent and possess strong immunomodulatory functions which could modulate various immune disorders (Li and Hua 2017). Previous studies have reported that SLE BMMSCs exhibit senescent characteristics and play a crucial role in SLE, and reversing the senescent phenotype of BMMSCs can improve the therapeutic effects of SLE (Dong et al. 2019; Li et al. 2012). Increasing evidence indicated that the imbalance between T-helper cells (Tfh) and regulatory T-cells (Treg) contributes to the development of SLE (Lee 2018; Noack and Miossec 2014). Meanwhile, the pathogenesis of SLE involves an acquired deficiency of cytokine IL-2, an essential growth and survival factor for Treg, which plays an important role in the control of autoimmunity in SLE (Humrich and Riemekasten 2016).

lncRNAs are a group of non-coding RNAs that is more than 200 nt and may have their own promoters and always lie between protein-coding genes (intergenic region) (Guttman et al. 2009). LncRNAs have been reported to affect the immune escape through regulating Treg cell differentiation. For instance, lncRNA NKILA promotes tumor immune evasion by sensitizing T cells to activation-induced cell death (Huang et al. 2018). LncRNA-MEG3 functions as a competing endogenous RNA to regulate Treg/Th17 balance in patients with asthma by targeting the microRNA-17/RORγt axis (Qiu et al. 2019). LncRNA H19 has been identified to play important roles in carcinogenesis and might be a candidate for the development of promising therapeutic and diagnostic modalities for several cancers (Yoshimura et al. 2018). However, the role of H19 in SLE has not been studied. In addition, it has been reported that the imbalance between Treg and Tfh cells is a characteristic of autoimmune diseases and is dependent on homeostatic cytokines, including IL-2 (Kosmaczewska 2014).

In this study, we found that H19 expression is significantly upregulated during SLE progression. H19 efficiently inhibits the proliferation, migration and induces apoptosis of BMMSCs. Meanwhile, H19 inhibits Treg cell proliferation and promotes the conversion of Treg cells to Tfh cells by inhibiting IL-2 production in SLE. Our results contribute to understanding the complex mechanism of SLE and provide a novel therapeutic target for SLE.

## Methods

### Subject

A total of 30 SLE patients, including 18 active SLE and 12 inactive SLE patients, and 30 healthy controls were recruited in this study. Active SLE was defined as a SLEDAI-2K score > 10, whereas those patients with SLEDAI-2K ≤ 10 were classed as relatively inactive SLE (Wu et al. 2015; Hayakawa et al. 2005). In addition, 22 patients with primary Sjögren’s syndrome (SS) were recruited as the disease control. Moreover, 9 other types of connective tissue diseases, including rheumatoid arthritis (RA) and dermatomyositis (DM), were used in the analysis. All participants provided written consent, and the study was approved by the Ethics Committee of Shenzhen Futian Hospital.

### Cell isolation and culture

BMMSCs were isolated from SLE patients and normal controls as previously described (Dong et al. 2019). Cells were cultured in low glucose Dulbecco’s Modified Eagle’s Medium (L-DMEM) (Gibco, USA) supplemented with 10% heat-inactivated fetal bovine serum (FBS) (Invitrogen, USA) and 1% antibiotic–antimycotic solution for adherent screening culture at 37 °C in a humidified incubator with 5% CO_2_. Medium containing non-adherent cells was replaced after 48 h and then every 3 days. Cells grown to 90% confluency were recovered with 0.25% trypsin-ethylenediaminetetraacetic acid (EDTA) (Gibco, USA) and seeded into 6-plate wells at a density of 1 × 10^6^ per 25 cm^2^ (Geng et al. 2019). Then cells were collected for the subsequent analysis.

### qRT-PCR

Total RNA was extracted from serum and BMMSCs of SLE patients or healthy controls using TRIzol reagent. Approximately 1 μg of RNA was reverse transcribed into complementary DNA (cDNA) using Superscript II reverse transcriptase. qRT-PCR was performed by Applied Biosystems 7500 Real-Time PCR System (Applied Biosystems, Foster City, CA). Primers used in this study were H19 forward TGGAGTCTGGCAGGAGTGATG and reverse CCAAAAGTGACCGGGATGAATG, GAPDH forward ACAGTCAGCCGCATCTTCTT and reverse R-GACAAGCTTC CCGTTCTCAG. Relative H19 mRNA expression was calculated with the comparative threshold cycle (Ct) (2^−ΔΔCt^) method (Schmittgen and Livak 2008), with *gapdh* as the internal reference.

### ELISA assay

The active and total IL-2 production was detected with the specific ELISA kit according to the manufacturers’ instructions.

### Cell transfection

The vectors including H19 negative control (H19-NC), the full‐length H19 sequence (H19-OE), and inhibitor-H19 (H19-KD) were synthesized and purchased from Invitrogen. Transfection in BMMSCs was performed using Lipofectamine 3000. After transfection for 48 h, cells were collected for the subsequent experiments. H19-KD sequence was 5′-GCCCGGGCTAGGACCGAGGAG-3′.

### Co-culture of BMMSCs with peripheral blood mononuclear cells (PBMCs)

PBMCs were isolated as previously described (Chen et al. 2017). PBMCs were co-cultured with or without transfected BMMSCs for 72 h at a ratio of 10:1 in 96-well flat-bottomed plates in 200 μl RM1640 medium supplemented with soluble anti-human CD3 (1 μg/ml) and anti-human CD28 (1 μg/ml) antibodies. Recombinant human IL-2 or anti-human IL-2 antibody (10 μg/ml) was added for subsequent analysis.

### Differentiation assay

PBMCs were isolated from peripheral blood using Ficoll density-gradient centrifugation. Treg cells were obtained as CD4^+^CD25^−^T cell subsets, and naïve CD4^+^ T cells were isolated and purified using a naïve CD4^+^ T cell isolation kit (Miltenyi Biotec, Bergisch Gladbach, Germany) according to the manufacturer’s instructions. Then, CD4^+^CD25^−^ T cells (1 × 10^6^/well) were cultured with soluble anti-CD3 (1 μg/ml) and anti-CD28 (1 μg/ml) antibodies, with the addition of recombinant human TGF-β1 (10 ng/ml; R&D Systems, USA) and IL-2 (100 U/ml; Peprotech, USA) to induce Treg cell conversion. After culturing for 5–6 days, the cells were collected for the measurement of CD4^+^CD25^+^ percentages by flow cytometry.

Tfh cells were obtained as naive CD4^+^ T cells (1 × 10^6^/well) and stimulated with soluble anti-CD3 (1 μg/ml) and anti-CD28 (1 μg/ml) antibodies, with the addition of recombinant human IL-2 (100 U/ml; Peprotech, USA), IL-6 (20 ng/ml; Peprotech, USA), anti-IL-4 (10 μg/ml; R&D Systems, USA), anti-IFN-γ (10 μg/ml; R&D Systems, USA) and anti-TGF-β (10 μg/ml; R&D Systems, USA). After 5–6 days, the cells were collected for the measurement of CXCR5^++^PD-1^++^CD4^+^ T cell or CXCR5^+ +^PD-1^++^ Foxp3^+^CD4^+^ T cell percentages by flow cytometry.

### Dual-luciferase reporter assay

Partial H19 fragment containing the corresponding wild-type or mutant IL-2 binding site was amplified by PCR and subcloned into psiCHECK-2 luciferase reporter vector (Promega, Madison, WI, USA) to generate H19-WT and H19-MUT reporter plasmids. The reporter plasmids were then co-transfected with control or IL-2 into BMMSCs. The luciferase activities in cell lysates were detected 48 h after transfection by dual luciferase reporter assay kits (Promega).

### DAPI staining assay

Cells were plated into 96-well plates and cultured with a complete medium containing 50 µM 5-Ethynyl-2′-deoxyuridine (EdU) for 2 h. After washed with PBS, cells were fixed in 4% polyoxymethylene and treated with 0.5% Triton X-100 for 5 min and then incubated with an Apollo dye for another 30 min. Subsequently, the cells were rinsed with 0.5% Triton X-100 and methanol three times and stained with DAPI for the subsequent analysis.

### Apoptosis assay

BMMSCs (1 × 10^6^/well) transfected with H19-NC, H19-OE and H19-KD were stained using Annexin V/7AAD apoptosis detection kit. After culturing for 4–5 days, cells were detected by flow cytometry and then analyzed by using Flow-JO v 10.0.7.

### Migration assay

The migration assay was performed as previously described (Cruz-Mosso et al. 2018). Briefly, 1 × 10^5^ cells were plated into the upper chambers with serum-free media. The lower chambers were filled with media containing 20% FBS. After incubation for 24 h, cells on the bottom membranes were fixed by 4% paraformaldehyde and stained with DAPI. The migrated cells were photographed and counted under a microscope.

### Statistical analysis

All data were presented as the mean ± SEM. Data were calculated using GraphPad Prism 7.0 software. Differences between 2 groups were analyzed using an unpaired t test. One-way AVONA test was used to compare multiple groups. P < 0.05 was used as the significant threshold.

## Results

### H19 expression was upregulated in the serum and BMMSCs of SLE patients

**Fig. 1:**
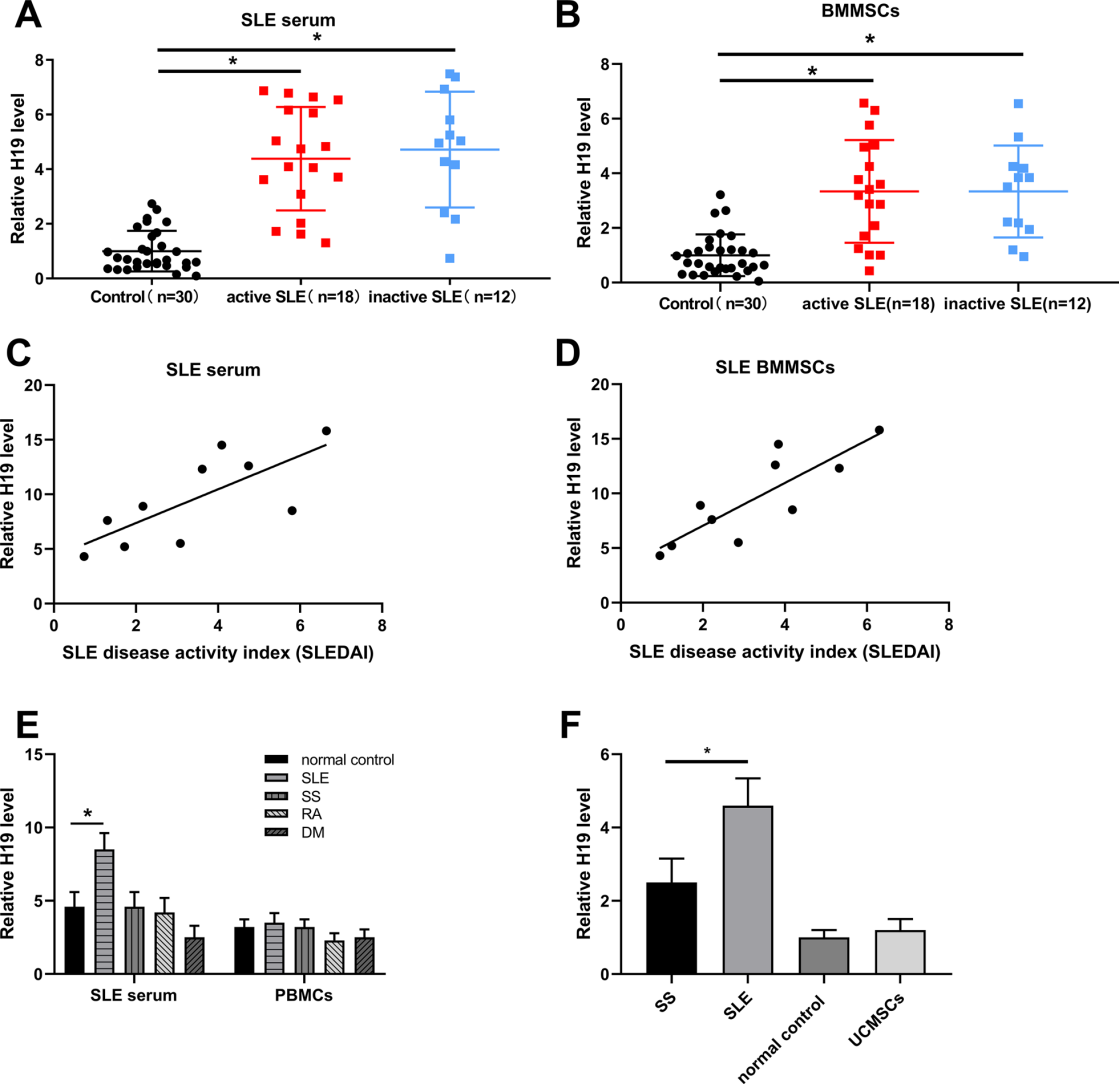
LncRNA H19 was upregulated during SLE. **A** and **B** H19 mRNA level in the serum (**A**) and BMMSCs (**B**) of SLE patients (n = 30) was detected by RT-qPCR. **C** and **D** Association of H19 expression in serum (**C**) and BMMSCs (**D**) with SLEDAI score. **E** H19 mRNA level in serum and PBMCs from SLE patients (n = 30), Sjögren's syndrome (SS) patients (n = 22), rheumatoid arthritis (RA) patients (n = 9), dermatomyositis (DM) patients (n = 9), and normal control was detected by RT-qPCR. **F** H19 mRNA level in BMMSCs from SS patients, SLE patients, normal controls, and umbilical cord-derived MSCs (UCMSCs) was detected by RT-qPCR. *P < 0.05

To explore the role of H19 in SLE progression, we detected H19 expression in the serum and BMMSCs of SLE patients (n = 30). The results indicated that H19 was highly expressed in SLE serum (Fig. 1A) and BMMSCs (Fig. 1B) compared with normal controls (p < 0.05). Moreover, we found that H19 mRNA level in both SLE serum (Fig. 1C) and SLE BMMSCs (Fig. 1D) was positively correlated with SLE disease activity index (SLEDAI). In addition, H19 mRNA level was also increased in SLE serum (p < 0.05) (Fig. 1E) but not in SLE PBMCs (Fig. 1F). In addition, H19 expression was lower in Sjögren's syndrome (SS) BMMSCs than SLE BMMSCs (p < 0.05) (Fig. 1G). These results indicated that H19 was potentially and positively related to the SLE progression.

### H19 inhibited proliferation and migration but promoted apoptosis of BMMSCs

**Fig. 2:**
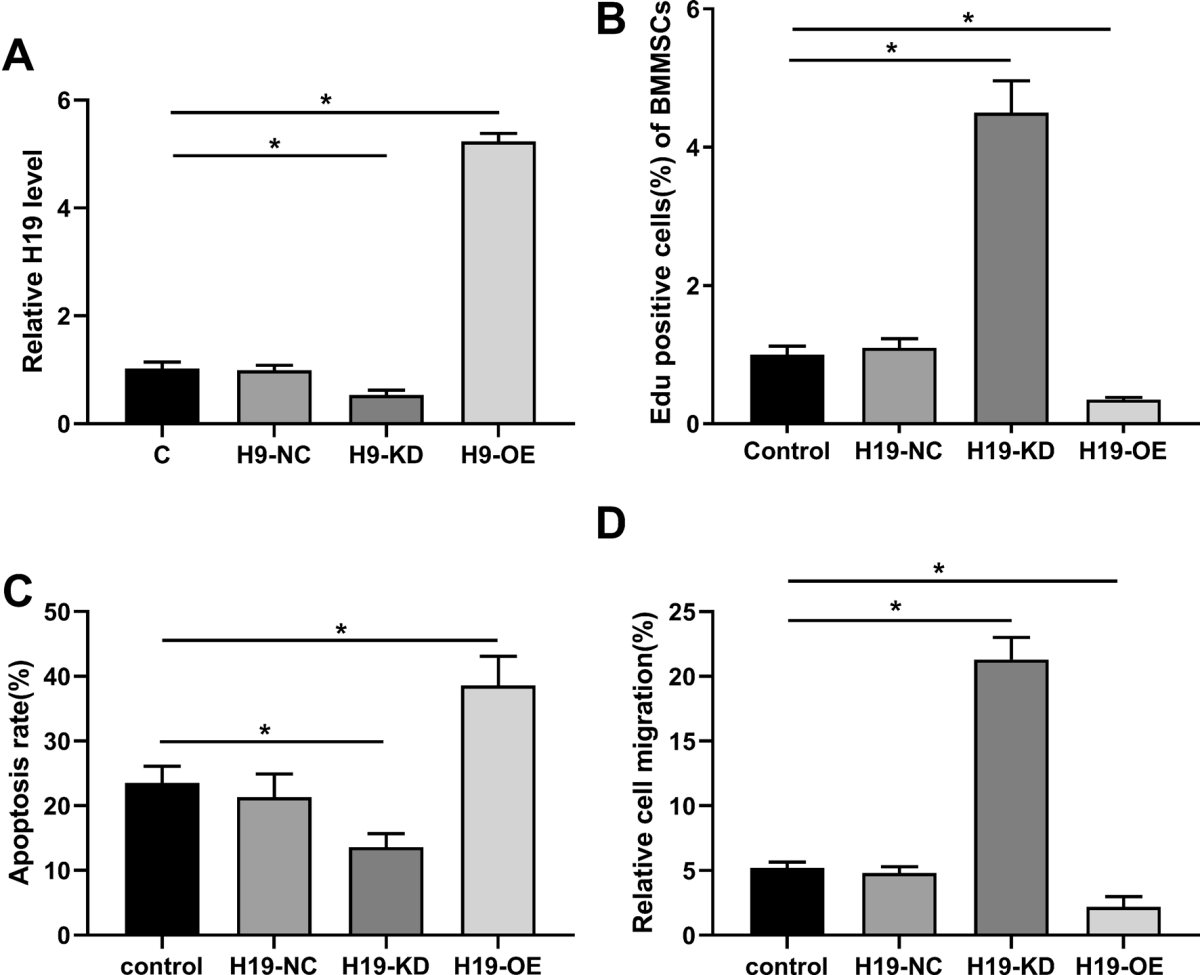
H19 inhibited proliferation and migration and promoted apoptosis of BMMSCs in vitro. The BMMSCs were transfected with H19-NC, H19-OE or H19-KD, and normal BMMSCs as control. **A** H19 level was measured by qPCR at 24 h of post-transfection to confirm the success of transfections. **B** Proliferation of cells in these four groups were measured by the EdU assay. **C** Apoptotic rate of cells in these four groups. **D** Migration ability of BMMSCs in these four groups were detected by migration assay. n = 6. *P < 0.05, **P < 0.01

To explore the role of H19 in BMMSCs, mimic-H19 (H19-OE), inhibitor-H19 (H19-KD) and negative control H19-NC were generated and transfected into BMMSCs. At 24 h after transfection, H19 level was significantly increased in H19-OE group but decreased in H19-KD group compared with H9-NC and C groups (p < 0.05) (Fig. 2A). Compared with H19-NC group, EdU positive BMMSCs were decreased in the H19-OE group but increased in the H19-KD group (p < 0.05) (Fig. 2B), suggesting that H19-OE significantly suppressed BMMSCs proliferation. Apoptosis analysis showed that H19-OE significantly promoted BMMSCs apoptosis rate, and H19-KD could remarkedly inhibited cell apoptosis (p < 0.05) (Fig. 2C). Meanwhile, migration assay indicated that H19-OE decreased BMMSC migration and H19-KD significantly increased BMMSC migration (p < 0.05) (Fig. 2D). These results indicated that H19 could significantly inhibit proliferation and migration and promote apoptosis of BMMSCs.

### H19 inhibited the immunoregulatory function of BMMSCs

**Fig. 3:**
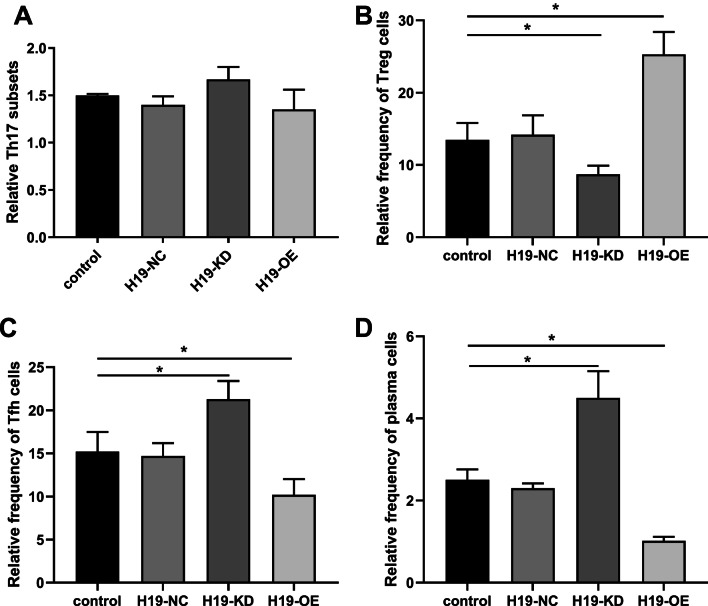
H19 inhibited the immunoregulatory function of BMMSCs. BMMSCs from SLE patients were transfected with H19-NC, H19-OE or H19-KD and then co-cultured with PBMCs at a ratio of 1:10 for 3 days. **A** Percentages of Th17 cells. **B** The proportion of Treg cells. The proportion of Tfh cells (**C**) and plasma cells (**D**). n = 6. *P < 0.05

To further explore whether H19 could affect the immunoregulatory function of BMMSCs, BMMSCs transfected with mimic-H19 (H19-OE), inhibitor-H19 (H19-KD) and negative control H19-NC were co-cultured with PBMCs. Compared with H19-NC group, there was no significant change in Th17 subsets with H19-OE or H19-KD transfected BMMSCs (p < 0.05) (Fig. 3A). In addition, the proportion of Treg cells was downregulated in H19-OE group and upregulated in H19-KD group (p < 0.05) (Fig. 3B), while the proportion of Tfh cells (Fig. 3C) and plasma cells (Fig. 3D) were upregulated in H19-OE group and downregulated in H19-KD transfected BMMSCs (p < 0.05). These data suggested that H19 regulated BMMSCs-mediated balance of Tfh/Treg cell.

### H19 interfered with BMMSCs-mediated growth and differentiation of Treg cells

**Fig. 4:**
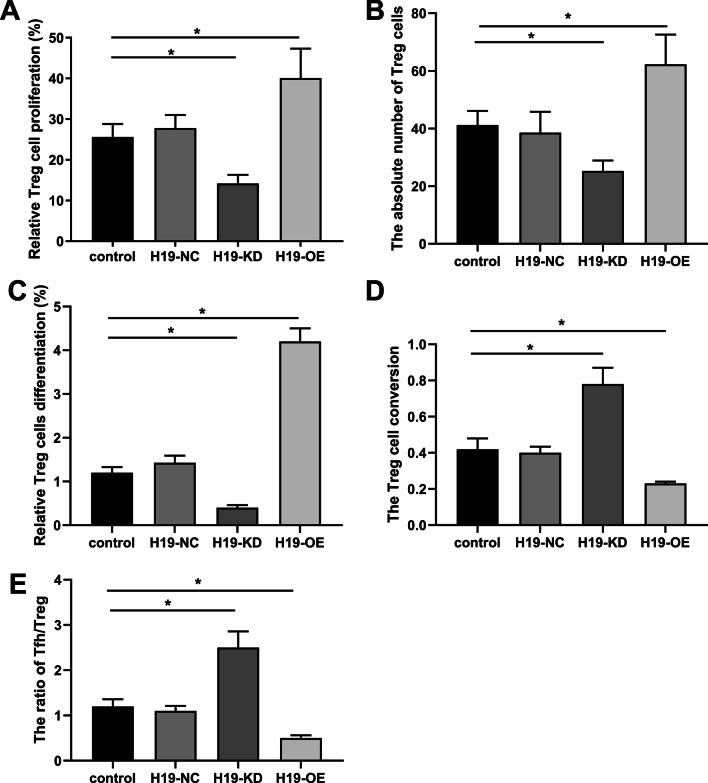
H19 inhibited Treg cell proliferation and promoted its differentiation to Tfh cells. BMMSCs were transfected with H19-NC, H19-OE or H19-KD and then co-cultured with Treg cells at a ratio of 1:10 for 3 days. H19-OE suppressed Treg cell proliferation (**A**) and absolute number (**B**). **C** H19-OE transfected BMMSCs decreased Treg cell differentiation. **D** H19-OE promoted the conversion of Treg cells to Tfh cells. **E** The ratio of Tfh/Treg was increased in the H19-OE. n = 6. *P < 0.05

To explore how H19 in BMMSCs interferes with the balance of Treg/Tfh cells, we co-cultured transfected BMMSCs with Treg cells. Compared with the control group, the proliferation (Fig. 4A) and absolute number (Fig. 4B) of Treg cells were elevated in the H19-KD group but reduced in the H19-OE group compared with H19-NC group (p < 0.05), suggesting that H19 inhibited BMMSCs-mediated Treg cells growth. Meanwhile, our results revealed that H19-KD transfected BMMSCs significantly increased the differentiation of Treg cells, while H19-OE transfected BMMSCs decreased Treg cell differentiation (p < 0.05) (Fig. 4C). As expected, H19-OE significantly promoted the conversion of Treg cells to Tfh cells, while H19-KD group decreased the conversion (p < 0.05) (Fig. 4D). Consequently, the ratio of Tfh/Treg was significantly increased in the H19-OE group but decreased in the H19-KD group (p < 0.05) (Fig. 4E).

### H19 directly inhibited IL-2 transcription in BMMSCs

**Fig. 5:**
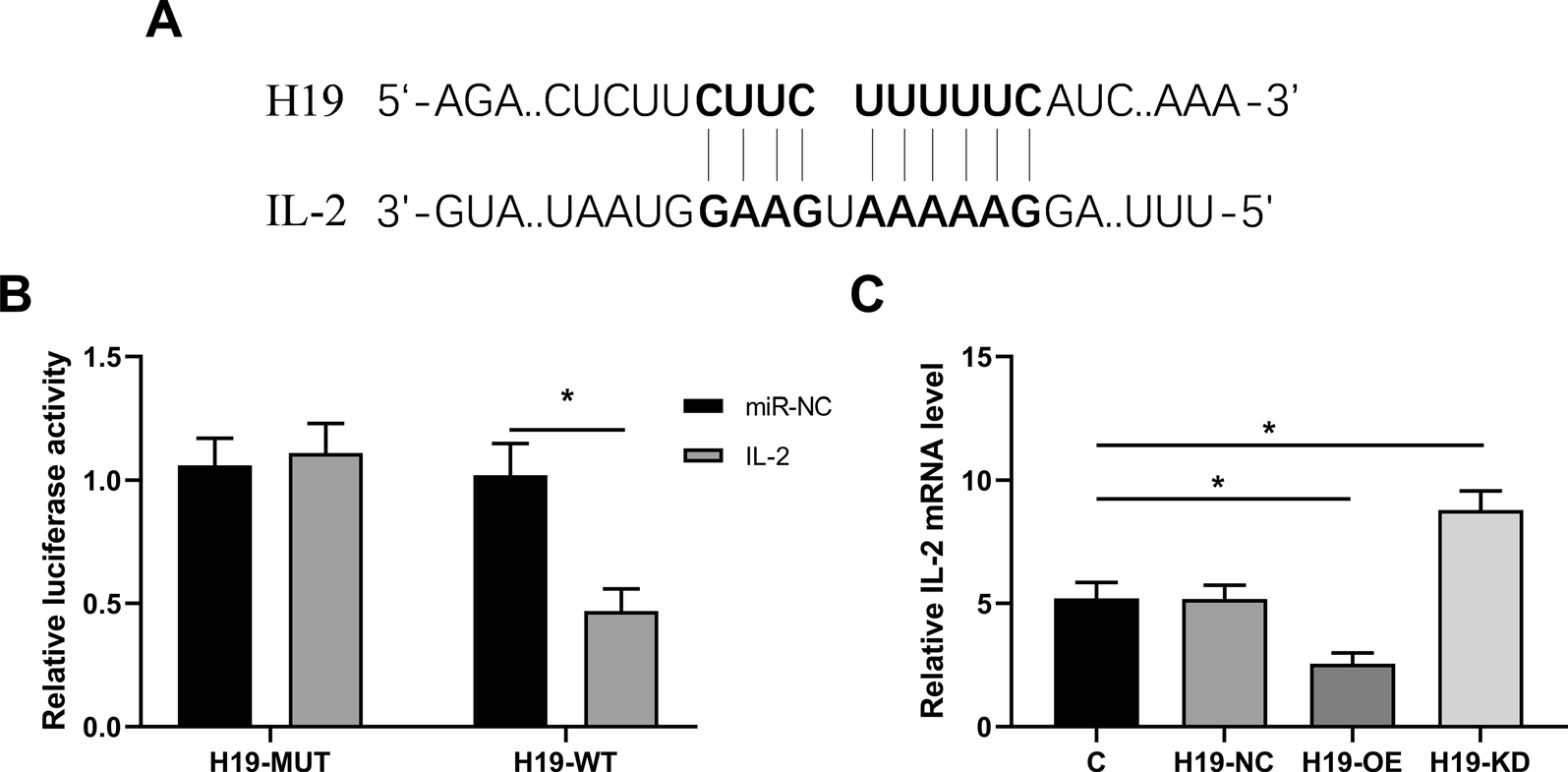
H19 inhibited directly IL-2 transcription in BMMSCs. **A** The interaction between H19 and IL-2 was predicted by IntaRNA. **B** Luciferase-reporter assay indicated H19 bound with IL-2. **C** The effects of H19 on IL-2 mRNA level was analyzed by qPCR. *P < 0.05

The interaction between H19 and IL-2 was predicted by IntaRNA (Qiu et al. 2019). A strong base-pairing was observed between H19 and IL-2 in in silico model (Fig. 5A). Dual luciferase activity assay revealed that the luciferase activity of H19-Wt was decreased in BMMSCs cells after IL-2 overexpression (p < 0.05) (Fig. 5B). In addition, our study showed that H19-OE reduced IL-2 mRNA level, while H19-KD increased IL-2 mRNA level (p < 0.05) (Fig. 5C).

### IL-2 was involved in the role of H19 in BMMSCs

**Fig. 6:**
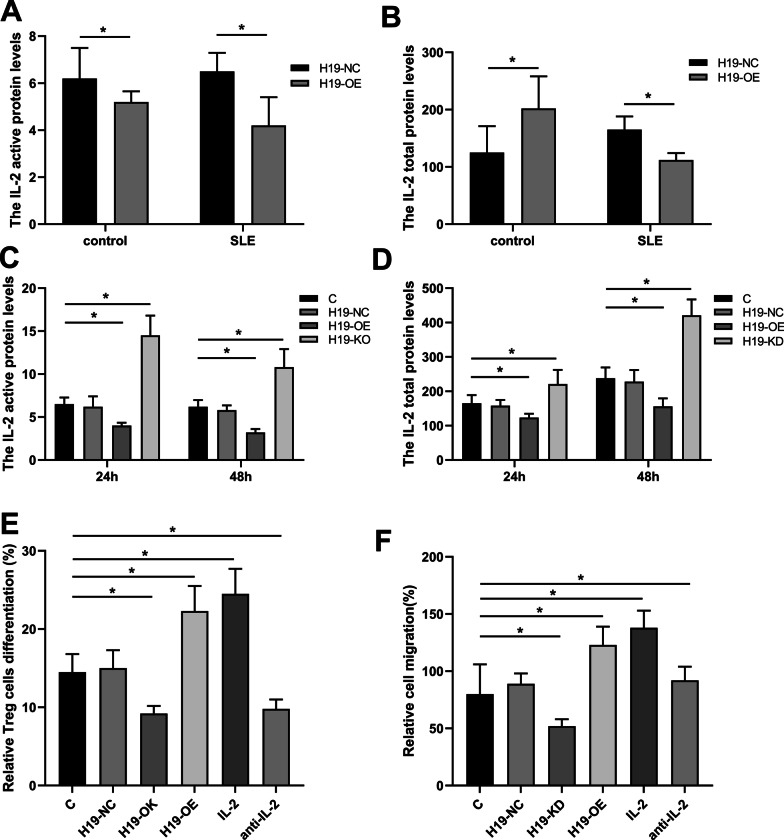
IL-2 was involved in the role of H19. **A** and **B** The level of active IL-2 (**A**) and total IL-2 (**B**) in cultured supernatants of BMMSCs from SLE patients and health controls was detected by ELISA assay. **C**–**F** BMMSCs were transfected with or without H19-NC, H19-OE or H19-KD and co-cultured with PBMCs for 24 h and 48 h. **C** and **D** The level of active IL-2 (**C**) and total IL-2 (**D**) in cultured supernatants of BMMSCs was detected by ELISA. **E** and **F** The role of H19 in the immunoregulatory function of BMMSCs on Treg cells (**E**) and migration (**F**) in medium supplemented with additional IL-2. *P < 0.05

To explore the specific mechanism of H19, we investigated whether H19 functions through IL-2, a key protein involved in the proliferation and differentiation of BMMSCs. We found that both active (p < 0.05) (Fig. 6A) and total (p < 0.05) (Fig. 6B) IL-2 protein levels were significantly lower in SLE BMMSCs than in controls at 24 h of co-culture. The active (Fig. 6C) and total IL-2 protein levels (Fig. 6D) were elevated in the H19-KD group and reduced in the H19-OE group at 24 h and 48 h (p < 0.05). To further confirm the role of IL-2, exogenous human recombinant IL-2 and anti-IL-2 antibody were applied in the BMMSCs-PBMCs co-culture system. The results showed that IL-2 could significantly enhance Treg cell differentiation (Fig. 6E) and migration (Fig. 6F) of BMMSCs caused by H19-OE in BMMSCs-PBMCs co-culture system (p < 0.05), and this effect was reversed by anti-IL-2 antibody. These results indicated that H19 induced immune dysregulation of BMMSCs by inhibiting IL-2 production.

## Discussion

Previous studies suggest that autoimmune diseases may be described as a stem cell disorder. Perez-Simon et al. reported that BMMSCs from chronic primary immune thrombocytopenia (ITP) patients showed an impaired proliferative capacity compared with those from normal controls (Perez-Simon et al. 2009). BMMSCs in SLE showed evidence of growth retardation in vitro (Sun et al. 2007). SLE is also a common autoimmune disease with IL-2 deficiency (He et al. 2016). It has been reported that BMMSCs from SLE patients exhibited early signs of senescence, which may participate in the development of SLE (Ji et al. 2017). These reports indicated that BMMSCs dysregulation was responsible for SLE.

LncRNA H19 has been reported to be closely involved in various human diseases. For instance, H19 promotes atherosclerosis by regulating MAPK and NF-kB signaling pathways (Pan 2017). H19 enhances skeletal muscle insulin sensitivity in part by targeting AMPK pathway Geng et al. 2018). H19 increases gefitinib resistance via packaging into exosomes in non‑small cell lung cancer (Lei et al. 2018). H19 magnifies the proliferation of pulmonary artery smooth muscle cells through AT1R via sponging let-7b in monocrotaline-induced pulmonary arterial hypertension (Su et al. 2018). H19 regulates the expression of its target gene HOXA10 in endometrial carcinoma by competing with miR-612 (Zhang et al. 2018a). H19 modulates cardiomyocyte apoptosis and acute myocardial infarction by targeting miR-29b (Yu and Dong 2018). In addition, H19 also affects a series of other human diseases, including pulmonary fibrosis, retinoblastoma, breast cancer, colorectal adenocarcinoma, coronary artery disease, acute myelocytic leukemia, and so on (Lu et al. 2018; Qi et al. 2019; Zhou et al. 2017; Li et al. 2018; Xiong and Jiang 2019; Zhao et al. 2017). These reports confirmed the important role of H19 and attracted us to focus on the effect of H19 in SLE. Here, we found that H19 expression was significantly upregulated in SLE serum and BMMSCs. Based on the important function of BMMSCs in SLE, we explore the role of H19 in BMMSCs. The results indicated that H19 significantly inhibited proliferation and migration and promoted apoptosis of BMMSCs, which account for the development of SLE.

T-follicular helper (Tfh) cells are a specialized subset of T cells that provide help to B cells and promote the formation of germinal centers (GCs). The balance between Treg cells and Tfh cells is crucial for the autoimmune in vivo (Qiu et al. 2017). Previous reports showed a significant increase in the proportion of circulating Tfh-like cells in patients with SLE, and circulating Tfh cells are positively associated with disease activity in SLE (Xu et al. 2015; Choi et al. 2015; Zhang et al. 2015). We further explored the role of H19 in the balance of Treg cells and Tfh cells. Interestingly, we found that H19 significantly suppressed BMMSCs-mediated Treg cells growth and enhanced the conversion of Treg cells to Tfh cells, and these effects were reversed by H19 inhibitor.

As we know, IL-2 is a crucial growth and survival factor for regulatory T cells (Treg) and the maintenance of immunological tolerance (Arenas-Ramirez et al. 2015). Recent studies showed that mice deficient in IL-2 or IL-2 receptor components, including CD25 and CD122, succumb to a rapidly progressing autoimmune disease characterized by uncontrolled activation of T and B cells and by generating autoantibodies (Suzuki et al. 1995), which was consistent with typical immunological features of SLE. We also confirmed that IL-2 production was decreased during SLE. Moreover, we demonstrated that H19 overexpression significantly decreased both active and total IL-2 levels, while H19-inhibitor could remarkedly increase IL-2 production, indicating that H19 plays an important role in regulating IL production.

IL-2 therapy has potential values to treat immune-mediated diseases. For example, Hartemann et al. has defined a well-tolerated and immunologically effective dose range of IL-2 for application to type 1 diabetes therapy and prevention (Hartemann et al. 2013). Low dose IL-2 increased regulatory T cells and elevated platelets in a patient with immune thrombocytopenia (Zhang et al. 2018b). In addition, IL-2 has also been applied for SLE treatment. He et al. demonstrated that low-dose recombinant human IL-2 selectively modulated the abundance of regulatory T (Treg) cells, follicular helper T (TFH) cells and IL-17-producing helper T (TH17) cells in patients with SLE (He et al. 2016). Here, we found that H19 significantly suppressed BMMSCs-mediated Treg cells growth and enhanced the conversion of Treg cells to Tfh cells. Meanwhile, additional IL-2 significantly promoted Treg cells caused by H19-OE. All these results support that H19 promotes SLE progression through inhibiting IL-2 production, at least in part. Our study revealed a new mechanism in SLE and contributed to identify and develop new treatment targets for SLE.

## Conclusion

In summary, our study demonstrated that lncRNA H19 could induce immune dysregulation of BMMSCs in SLE through inhibiting proliferation and migration and inducing apoptosis of BMMSCs, suppressing proliferation of Treg cells, and promoting the conversion of Treg cells to Tfh cells by directly inhibiting IL-2 production and provided novel treatment targets for SLE treatment.

## Data Availability

All data generated or analyzed during this study are included in this published article.
